# *DNMT1*, *DNMT3A* and *DNMT3B* Polymorphisms Associated With Gastric Cancer Risk: A Systematic Review and Meta-analysis

**DOI:** 10.1016/j.ebiom.2016.10.028

**Published:** 2016-10-19

**Authors:** Hongjia Li, Wen Li, Shanshan Liu, Shaoqi Zong, Weibing Wang, Jianlin Ren, Qi Li, Fenggang Hou, Qi Shi

**Affiliations:** a Oncology Department of Shanghai Municipal Hospital of Traditional Chinese Medicine affiliated to Shanghai University of Traditional Chinese Medicine, Shanghai 200071, China; b Fudan University School of Public Health, Shanghai 200032, China; c Department of Medical Oncology, Shuguang Hospital, Shanghai University of Traditional Chinese Medicine, Shanghai 201203, China

**Keywords:** DNA methyltransferase, Single nucleotide polymorphism, Gastric cancer, Meta-analysis, Systematic review

## Abstract

**Background:**

Increasing studies showed that abnormal changes in single nucleotide polymorphisms (SNPs) of *DNMTs* (*DNMT1*, *DNMT3A* and *DNMT3B*) were associated with occurrence or decrease of various tumors. However, the associations between *DNMTs* variations and gastric cancer (GC) risk were still conflicting. We aimed to assess the effect of *DNMTs* polymorphisms on the susceptibility to GC.

**Methods:**

Firstly, we did a meta-analysis for 7 SNPs (rs16999593, rs2228611, rs8101866 in *DNMT1*, rs1550117, rs13420827 in *DNMT3A*, rs1569686, rs2424913 in *DNMT3B*). Four genetic models (homozygote, heterozygote, dominant and recessive model) were used. Moreover, a meta-sensitivity and subgroup analysis was performed to clarify heterogeneity source. Lastly, 17 SNPs that couldn't be meta-analyzed were presented in a systematic review.

**Findings:**

20 studies were included, 13 studies could be meta-analyzed and 7 ones could not. Firstly, a meta-analysis on 13 studies (3959 GC cases and 5992 controls) for 7 SNPs showed that GC risk increased in rs16999593 (heterozygote model: OR 1.36, 95%CI 1.14–1.61; dominant model: OR 1.36, 95%CI 1.15–1.60) and rs1550117 (homozygote model: OR 2.03, 95%CI 1.38–3.00; dominant model: OR 1.20, 95%CI 1.01–1.42; recessive model: OR 1.96, 95%CI 1.33–2.89) but decreased in rs1569686 (dominant model: OR 0.74, 95%CI 0.61–0.90). The remaining SNPs were not found associated with GC risk. Furthermore, the subgroup analysis indicated that for rs1550117 and rs1569686, the significant associations were particularly found in people from Chinese Jiangsu province (rs1550117, OR 1.77, 95%CI 1.25–2.51; rs1569686, OR 0.48, 95%CI 0.36–0.64) and that PCR-RFLP was a sensitive method to discover significant associations (rs1550117, OR 1.77, 95%CI 1.25–2.51; rs1569686, OR 0.49, 95%CI 0.37–0.65). Lastly, a systematic review on 7 studies for 17 SNPs suggested that rs36012910, rs7560488 and rs6087990 might have a potential effect on GC initiation.

**Conclusion:**

This meta-analysis demonstrated that rs16999593 and rs1550117 could contribute to GC risk and that rs1569686 might be a protective factor against gastric carcinogenesis. By using these SNPs as biomarkers, it is feasible to estimate the risk of acquiring GC and thus formulate timely preventive strategy.

## Highlights

• *DNMT1*, *DNMT3A* and *DNMT3B* polymorphisms are associated with gastric cancer (GC) risk, in which rs16999593 and rs1550117 might contribute to GC and rs1569686 could be a protective factor against carcinogenesis.
• Significant *DNMTs* polymorphisms were particularly found in people from Chinese Jiangsu province.
• PCR-RFLP was a sensitive method to discover significant *DNMTs* polymorphisms.

**Research in context** Genetic factors play a crucial role in GC risk. *DNMTs* genes were associated with occurrence or decrease of various tumors. But the effects of *DNMTs* on gastric cancer (GC) were not clear. Now our results proved that two and one variations in *DNMTs* were associated with GC, indicating a range of effects from the increased to the reduced. By using these variations as biomarkers, it is feasible to estimate the risk of acquiring GC and thus formulate timely preventive strategy.

## 1 Introduction

In 2012, 951,000 new gastric cancer (GC) cases and 723,000 deaths were estimated worldwide, making it the fifth most common tumor (Ferlay et al., 2015, Torre et al., 2015). GC is a complex disease arising from environmental and genetic factors. However in individuals infected with *H. pylori*, defined as a definite gastric carcinogen (Yang, 2006), only a few eventually develop into GC, which suggested that host genetic factors may play a crucial role in the susceptibility of GC (Saeki et al., 2013).

The epigenetics is believed to be important in the development of cancers, which was defined as a stably heritable changes through modifying gene expression without DNA sequence alterations (Esteller, 2008). The most common epigenetic phenomenon is DNA methylation that refers to a methyl group is conferred to the 5′ carbon of a cytosine in a CpG dinucleotide. It is catalyzed by a family of DNA methyltransferases (DNMTs) mainly consisting of three activated forms: DNMT1, DNMT3A and DNMT3B. DNMT1 is thought to be a maintenance DNA methyltransferase which principally maintains CpG methylation, involving in embryonic development and somatic cells survival (Brown and Robertson, 2007) and it is encoded by *DNMT1* gene which locates on chromosome 19p13.2 (Jiang et al., 2012a). DNMT3A and DNMT3B are considered as de novo methyltransferases which are required for the establishment of embryonic methylation patterns, mainly occurring during gametogenesis and early development (Okano et al., 1999) and they are encoded by *DNMT3A* and *DNMT3B* genes locating on chromosome 2p23 and 20q11.2 respectively (Yang et al., 2012).

There is considerable evidence that a number of abnormal changes in single nucleotide polymorphisms (SNPs) of *DNMTs* (*DNMT1*, *DNMT3A* and *DNMT3B*), which could cause DNA hypo-methylation or hyper-methylation (Gao et al., 2011, Fu et al., 2010, Harder et al., 2008, Zhao and Bu, 2012), are correlated to tumor occurrence or decrease (Luo et al., 2015, Chang et al., 2014, Mostowska et al., 2013, Kullmann et al., 2013, Sun et al., 2012, Xiang et al., 2010, Kanai et al., 2003) such as head and neck cancer, and colorectal cancer (Zhu et al., 2015, Duan et al., 2015). However, the associations between *DNMTs* SNPs and GC risk were still conflicting (Jiang et al., 2012a, Yang et al., 2012). Therefore, for the first time, the effects of *DNMTs* polymorphisms on the susceptibility to GC were systematically and comprehensively estimated.

## 2 Materials and Methods

### 2.1 Search

We did a literature search of PubMed, MEDLINE, Embase, Sinomed, CNKI, and WanFang databases to identify relevant studies up to June 1, 2016, using the search strategy: (stomach OR gastric) AND (neoplasms OR tumors OR cancers OR carcinomas) AND (DNMT1 OR DNMT3A OR DNMT3B OR DNMTs OR DNA methyltransferases). The languages were limited to English and Chinese. The search strategy for PubMed was listed in Appendix A.

### 2.2 Selection Criteria

All studies included in the meta-analysis were accorded with the following inclusion criteria: (a). study focused on the association of *DNMTs* polymorphisms and GC risk; (b). case-control or cohort studies. In addition, exclusion criteria were as follows: (a). reviews or meta-analysis; (b). overlapped articles or studies with overlapping data.

### 2.3 Data Extraction

Two investigators independently extracted the following data: first author, year of publication, province/country of origin, ascertainment of cases, source of controls, genotyping methods, *DNMT* genes, SNPs, number of cases and controls, and value of HWE. To ensure accuracy of the data, inconsistencies were discussed with another reviewer until reach a consensus.

### 2.4 Quality Assessment

The quality of each study was assessed according to the quality assessment criteria (Table S1) (Thakkinstian et al., 2011, Xue et al., 2015), in which the overall quality scores ranged from 0 to 15. Studies with scores ≥ 9 were regarded as high quality studies; otherwise, studies were considered to have a low quality.

### 2.5 Data Analysis

Stata software (version 12.0; Stata Corporation, College Station, TX) was used to perform all analysis. We used four types of genetic models (Lieb et al., 2006): homozygote model (homozygous rare vs. homozygous frequent allele), heterozygote model (heterozygous vs. homozygous frequent allele), dominant model (homozygous rare + heterozygous vs. homozygous frequent allele) and recessive model (homozygous rare vs. heterozygous + homozygous frequent allele). Association between *DNMTs* polymorphisms and the GC risk was evaluated by pooled odds ratios (OR), 95% confidence interval (95% CI) and *P* value of Z test (*P*_*OR*_). If 95%IC across 1 or *P*_*OR*_ < 0.05, a significant association existed. Then if OR or 95%IC < 1, the mutant gene was a protective factor; otherwise, it was a risk factor. Heterogeneity was analyzed using the *P* value of Q test (*P*_*het*_) and *I*^2^. If *P*_*het*_ < 0.1 or *I*^2^ > 50%, a significant heterogeneity existed. And then a sensitivity analysis and a subgroup analysis were performed. Sensitivity analysis was conducted through omitting one study by turns (Lu et al., 2016), if the 95%CI markedly deviated from the original interval or the *I*^2^ largely decreased, this study was an originator of heterogeneity.

## 3 Results

### 3.1 Literature Search and Study Characteristics

**Fig. 1:**
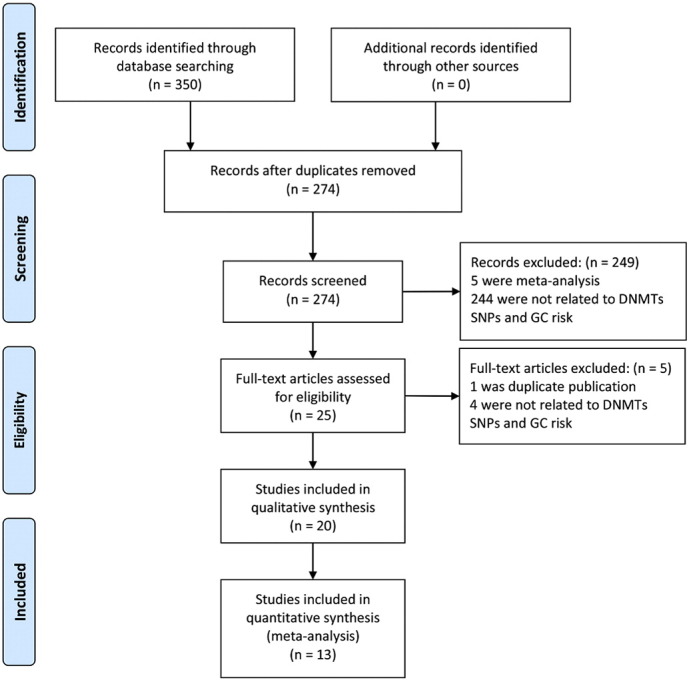
Flow chart of study selection process.

A total of 350 records were identified through database searching. After removing duplicates, 274 records were screened on details of the abstracts. In those 249 publications were excluded because 5 were meta-analysis and the other 244 were not related to *DNMTs* SNPs and GC risk. Then 25 full-text articles were obtained to be assessed, in which 5 articles were excluded because 1 was duplicate publication and 4 did not contain information on *DNMTs* SNPs and GC risk. Ultimately, 20 eligible studies (Jiang et al., 2012a, Yang et al., 2012, Yan et al., 2015, Khatami et al., 2009, Wu et al., 2014, Cao et al., 2013, Wu et al., 2012, Fan et al., 2010, Liu, 2009, Zhang et al., 2014, Hu et al., 2010, Zhang, 2008, Liu, 2008, Wang et al., 2005, Aung et al., 2005, Wang et al., 2015a, Jiang et al., 2013, Jiang et al., 2012b, Cao et al., 2012, Chang et al., 2010)were included in the qualitative synthesis, and 7 of them could not be quantitatively synthesized (3 studies respectively reported a different SNP (Wu et al., 2014, Wu et al., 2012, Liu, 2008), 4 studies were conference abstracts (Jiang et al., 2013, Jiang et al., 2012b, Cao et al., 2012, Chang et al., 2010)), so 13 studies involving 3959 GC cases and 5992 healthy controls were finally included in the meta-analysis (Fig. 1). Among the 20 studies, 18 studies were for Chinese population (respectively from Jiangsu, Jiangxi, Hebei, Shandong, Jilin and Heilongjiang provinces of China), 1 study was for Iranian population (from Fars and Tork) and another one was for Japanese population (from Hiroshima and Yamaguchi). According to the quality assessment criteria (Table S1), scores of the 13 studies (included in the meta-analysis) were 4–12 and 8 studies were with high quality scores (Xue et al., 2015). The main characteristics of the 13 studies were listed in Table 1.

### 3.2 Meta-analysis and Systematic Review

**Fig. 2:**
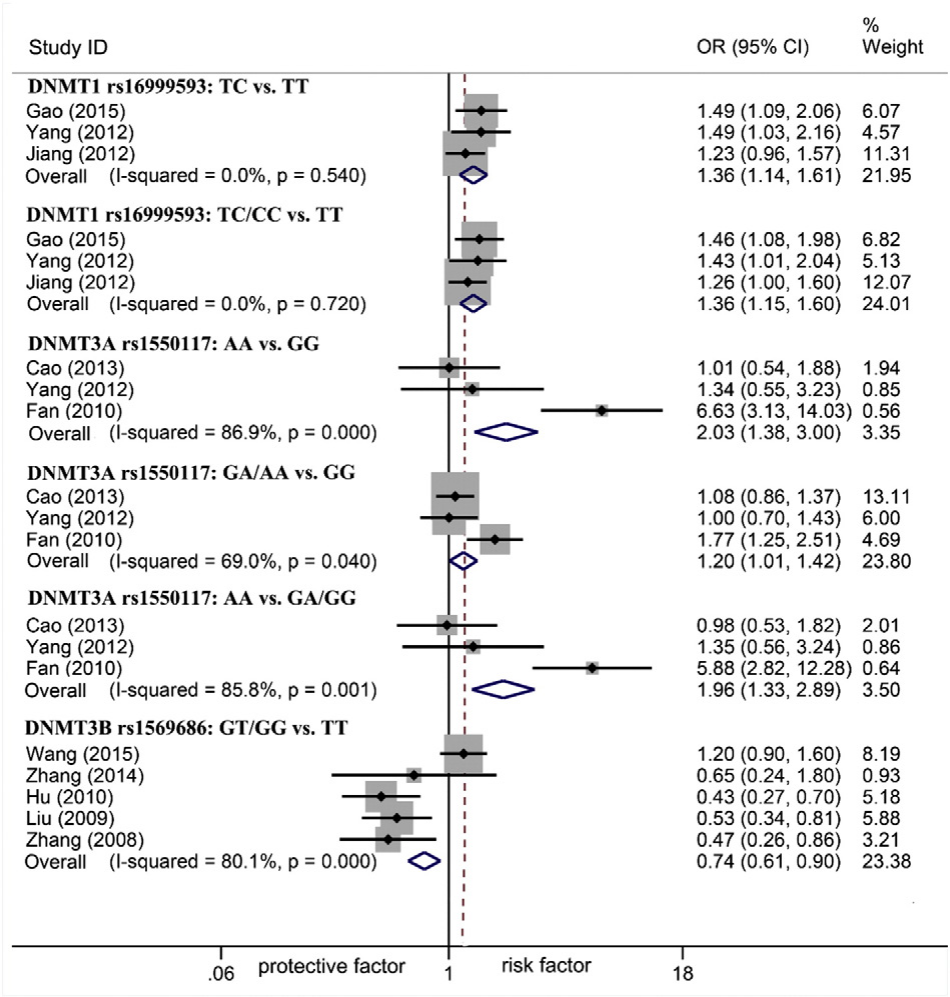
Forest plot of *DNMT1*, *DNMT3A* and *DNMT3B* polymorphisms associated with GC risk.

The associations between *DNMTs* polymorphisms and gastric carcinogenesis were shown in Table 2 and the statistically significant associations (only Chinese population were discovered in significant associations) were represented in Fig. 2. In terms of *DNMT1* and *DNMT3A*, GC risk increased. For rs16999593, there was an association under heterozygote and dominant models (TC vs. TT: OR 1.36, 95%CI 1.14–1.61; TC/CC vs. TT: OR 1.36, 95%CI 1.15–1.60) but not homozygote and recessive models (CC vs. TT: OR 1.36, 95%CI 0.93–1.99; CC vs. TC/TT: OR 1.22, 95%CI 0.84–1.78). For rs1550117, the increased GC risk was discovered under homozygote, dominant and recessive models (AA vs. GG: OR 2.03, 95%CI 1.38–3.00; GA/AA vs. GG: OR 1.20, 95%CI 1.01–1.42; AA vs. GA/GG: OR 1.96, 95%CI 1.33–2.89) but not heterozygote model (GA vs. GG: OR 1.12, 95%CI 0.93–1.33). Conversely, GC risk decreased in *DNMT3B*. For rs1569686, the association was found under dominant model (GT/GG vs. TT: OR 0.74, 95%CI 0.61–0.90) but not heterozygote, homozygote and recessive models (GT vs. TT: OR 0.88, 95%CI 0.69–1.13; GG vs. TT: OR 0.96, 95%CI 0.46–2.01; GG vs. GT/TT: OR 0.97, 95%CI 0.46–2.02). Except all of the above, for rs2228611, rs8101866, rs13420827 and rs2424913, no significant associations were observed among all of the genetic models. Lastly, for SNPs not able to be quantitatively synthesized, the systematic review presented their associations with GC (Table 3). Three SNPs rs36012910, rs7560488 and rs6087990 (Wu et al., 2014, Wu et al., 2012, Liu, 2008) were reported associated with GC and others not.

### 3.3 Heterogeneity Analysis (Sensitivity and Subgroup Analysis)

**Fig. 3:**
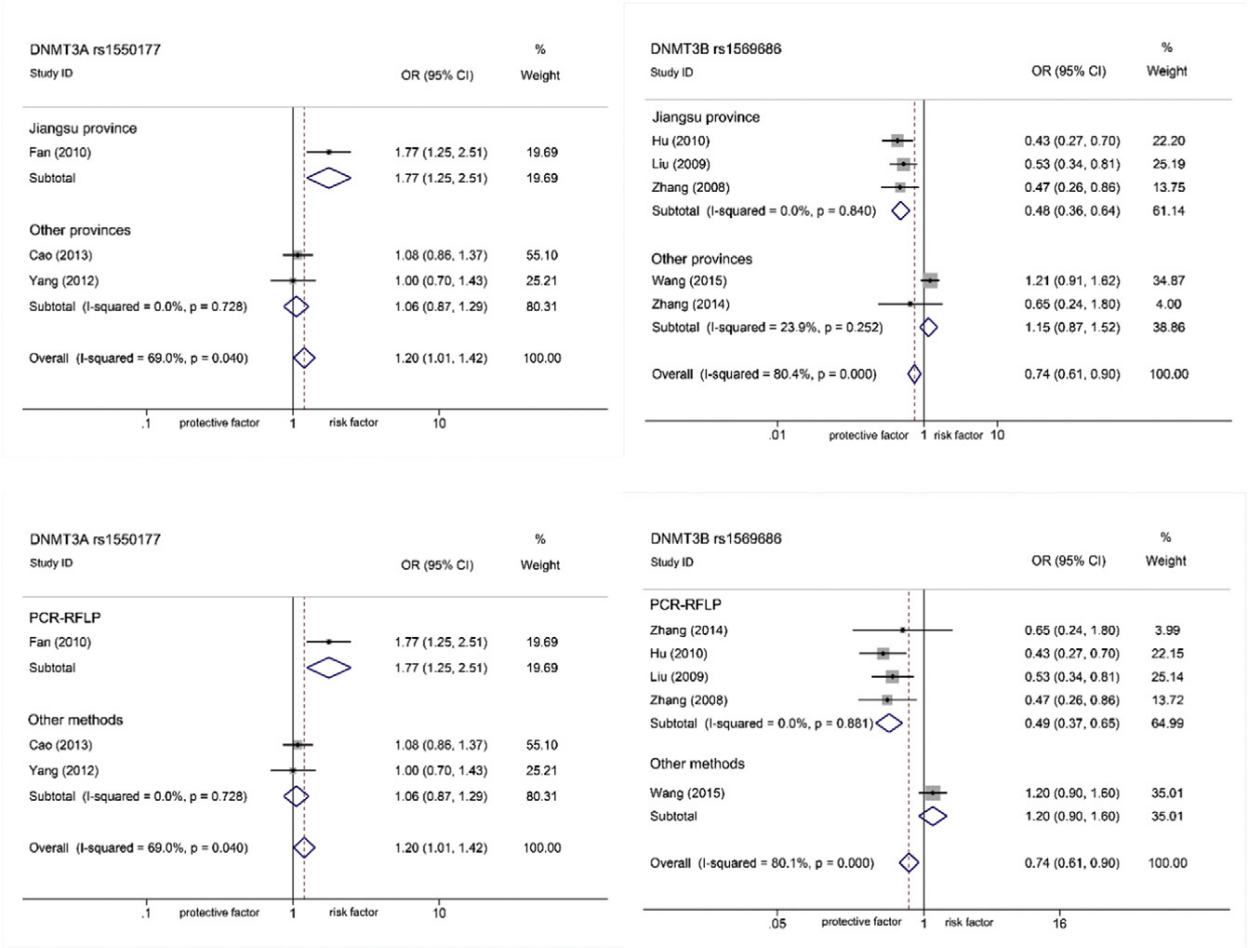
Forest plot of subgroup analysis on *DNMT3A* rs1550117 and *DNMT3B* rs1569686 polymorphisms (dominant model) by population area and genetic methods. Population area (Jiangsu province and other provinces: Jiangxi, Jilin and Heilong Jiang provinces, in China) (A); Genetic methods (PCR-RFLP and other methods: TaqMan and MassArray) (B).

There was obvious heterogeneity in rs1550117 (AA vs. GG *I*^2^ 86.9%, *P*_*het*_ 0.000; GA/AA vs. GG: *I*^2^ 69.0%, *P*_*het*_ 0.040; AA vs. GA/GG: *I*^2^ 85.8%, *P*_*het*_ 0.001) and rs1569686 (GT vs. TT: *I*^2^ 83.7%, *P*_*het*_ 0.002; GT/GG vs. TT: *I*^2^ 80.1%, *P*_*het*_ 0.000). A sensitivity analysis was conducted to explore which study primarily influenced the pooled ORs (Table S2, Fig. S1–S2). For rs1550117, the heterogeneity was mostly caused by a study (Fan et al., 2010), since when it was removed, 95%IC changed in direction of association (OR 1.06, 95%CI 0.87–1.29) and heterogeneity went to zero (*I*^2^ 0%, *P*_*het*_ 0.73). Likewise, for rs1569686, Wang et al. (2015b) was found to be the major originator after excluded (95%IC didn't change in direction but heterogeneity went to zero: OR 0.49, 95%CI 0.37–0.65, *I*^2^ 0%, *P*_*het*_ 0.88). We compared characteristics of the two studies to the other's. Two factors were screened out to explain the heterogeneity: population areas (Jiangsu province or others) and genotyping methods (PCR-RFLP or others). Then a subgroup analysis was performed (Fig. 3). Population areas: for Jiangsu population, rs1550117 and rs1569686 were associated with GC (OR 1.77, 95%CI 1.25–2.51; OR 0.48, 95%CI 0.36–0.64), but for others (Jiangxi, Jilin and Heilongjiang provinces) no associations were found (OR 1.06, 95%CI 0.87–1.29; OR 1.15, 95%CI 0.87–1.52). Genotyping methods: by PCR-RFLP, rs1550117 and rs1569686 were detected associated with GC (OR 1.77, 95%CI 1.25–2.51; OR 0.49, 95%CI 0.37–0.65) but by others (TaqMan and MassArray) significant associations were not discovered (OR 1.06, 95%CI 0.87–1.29; OR 1.20, 95%CI 0.90–1.60).

## 4 Discussion

Of the seven SNPs, two (rs16999593 and rs1550117) and one (rs1569686) were significantly associated with GC risk indicating a range of effects from the increased (*DNMT1* and *DNMT3A*) to the reduced (*DNMT3B*).

### 4.1 *DNMT1*

Our results proved rs16999593 as a potential biomarker for GC susceptibility which was exactly consistent with the results on other types of cancers, such as breast cancer and prostate cancer (Tao et al., 2015, He et al., 2014). In addition, we did not find rs2228611 associated with GC, but it was recently reported that patients carrying the mutant genotypes significantly lived longer than those bearing the wild, indicating that rs2228611 might be a positive prognostic marker for GC survival (Jia et al., 2016).

### 4.2 *DNMT3A* and *DNMT3B*

In terms of rs1550117, our findings opposed a previous meta-analysis and we could attribute this contradiction to differences in using homozygote models (Liu et al., 2015). For rs1569686, we consider it as a protective factor for gastric carcinogenesis and similar results were discovered in head and neck cancer, lung cancer and colorectal cancer (Duan et al., 2015, Zhang et al., 2015, Xia et al., 2015, Zhu et al., 2012). However, another study argued it was associated with poor prognosis in GC cases (Wang et al., 2015a). Maybe it played different roles in pathogenesis and prognosis. Particularly, we found in Jiangsu, a high GC incidence area of China (Liu et al., 2007), mutant rs1550117 doubled the risk and mutant rs1569686 lowered by a half of it. Also, even though some studies discovered TaqMan was more specific and sensitive than PCR-RFLP to detect polymorphisms or virus (Martinez-Trevino et al., 2016, Campsall et al., 2004), we found PCR-RFLP was so far a best method for risk detection in GC. Regarding rs2424913, we didn't find it associated with GC in Chinese. A review reported it could significantly decrease cancers in African but not Asian (Duan et al., 2015). It was speculated whether rs2424913 enabled African to catch GC rather than other populations. Although some meta-analysis studies demonstrated that rs6087990 might confer protection against overall cancers (Duan et al., 2015, Zhang et al., 2015), but it represented an opposite effect on GC as our systematic review showed (Liu, 2008).

### 4.3 Strengths and Limitations

Previous meta-analysis studies primarily evaluated associations between a few SNPs and cancers without classification, such as GC (Zhu et al., 2015, Duan et al., 2015, Liu et al., 2015, Zhang et al., 2015, Xia et al., 2015). The major strengths of our study was its comprehensive and systematic focus on GC and SNPs from three main types of *DNMTs*, 17 SNPs in total. Also, some mistakes in previous results were corrected in our study (Liu et al., 2015). At the same time, there were some limitations. Firstly, significant heterogeneities were observed for a few genetic models. Although a sensitivity analysis and a subgroup analysis were performed to clarify sources, we cannot find all potential factors. Second the meta-analysis findings were currently restricted to Chinese population pending results from other populations in future studies.

## 5 Conclusion

Our meta-analysis suggested that *DNMT1* rs16999593 and *DNMT3A* rs1550117 could contribute to GC and that *DNMT3B* rs1569686 might function as a protective factor against gastric carcinogenesis. By using these significant SNPs as biomarkers, it is feasible to estimate the risk of catching GC and thus formulate timely preventive strategy.

## Author Contributions

F.G.H., Q.S. and H.J.L conceived and designed the study. H.J.L., W.L., S.S.L. and S.Q.Z. took full responsibility for data collecting and accuracy. H.J.L. and W.L. performed the meta-analysis and systematic review, and drafted the manuscript. W.B.W., J.L.R., and Q.L. helped revise the manuscript.

## Funding

This work was supported by Natural Science Foundation of China (NSFC, NO: 81473624) and Key Specialty Foundation of The State Administration of Traditional Chinese Medicine (NO:ZJ0901ZL020). The sponsor had no role in study design, data collection, data analysis, data interpretation, or writing of the report.

## Conflicts of Interest

The authors have no conflicts of interest.

## Figures and Tables

**Table 1:** Characteristics of 13 studies included in the meta-analysis.

Study	Province/Country	Ascertainment of cases	Source of controls	Genotyping methods	Gene	SNPs	Sample size (cases/controls)	HWE (controls)	Score
Yan et al., 2015	Shandong/China	Histological	HB	Sequencing	*DNMT1*	rs16999593	310/420	0.469	9
						rs2228611		0.423	
Yang et al., 2012	Jiangxi/China	Histological	HB	MassArray	*DNMT1*	rs16999593	242/294	0.120	9
						rs2228611		0.068	
						rs8101866		0.747	
					*DNMT3A*	rs1550117		0.444	
						rs13420827			
Jiang et al., 2012a, Jiang et al., 2012b	Jilin/China	Histological	HB	TaqMan	*DNMT1*	rs16999593	447/961	0.910	9
						rs8101866			
Khatami et al., 2009	Fars/Iran, Tork/Iran	Histological	HB	PCR-RFLP	*DNMT1*	rs2228611	200/200	0.187	9
Cao et al., 2013	Jilin/China	Histological	HB	TaqMan	*DNMT3A*	rs1550117	447/961	0.658	9
						rs13420827		0.833	
Fan et al., 2010	Jiangsu/China	Histological	HB/PB	PCR-RFLP	*DNMT3A*	rs1550117	208/346	0.205	12
Liu, 2009	Jiangsu/China	NA	NA	PCR-RFLP	*DNMT3B*	rs2424913	308/189	0.942	6
						rs1569686	313/350	> 0.05	
Wang et al., 2015a, Wang et al., 2015b	Jilin/China	Histological	HB	TaqMan	*DNMT3B*	rs1569686	447/961	0.001	7
Zhang et al., 2014	Heilongjiang/China	NA	NA	PCR-RFLP	*DNMT3B*	rs1569686	50/60	0.389	4
Hu et al., 2010	Jiangsu/China	Histological	HB/PB	PCR-RFLP	*DNMT3B*	rs2424913	259/262	0.926	12
						rs1569686		0.901	
Zhang, 2008	Jiangsu/China	NA	HB	PCR-RFLP	*DNMT3B*	rs2424913	156/156	0.968	6
						rs1569686		0.001	
Wang et al., 2005	Hebei/China	Histological	HB/PB	PCR-RFLP	*DNMT3B*	rs2424913	212/294	0.654	12
Aung et al., 2005	Hiroshima/Japan, Yamaguchi/Japan	Histological	HB	PCR-RFLP	*DNMT3B*	rs2424913	152/247	1.000	6

NA, not available; HB, hospital based; PB, population based; PCR-RFLP, polymorphism chain reaction-restriction fragment length polymorphism; *DNMT genes*, deoxyribonucleic acid methyltransferase genes; SNPs, single nucleotide polymorphisms; HWE, Hardy-Weinberg equilibrium.

**Table 2:** Meta-analysis of association between *DNMTs* SNPs and gastric cancer risk.

SNPs	N (cases/controls)	OR (95%CI)	*P*_*OR*_^a^	*I*^2^	*P*_*het*_^b^
*DNMT1* rs16999593					
TC vs. TT^c^	949/1609	**1.36 (1.14,1.61)**	**0.001**	**0.0%**	**0.540**
CC vs. TT^d^	654/1202	1.36 (0.93,1.99)	0.117	0.0%	0.743
TC/CC vs. TT^e^	999/1675	**1.36 (1.15,1.60)**	**0.000**	**0.0%**	**0.720**
CC vs. TC/TT^f^	999/1675	1.22 (0.84,1.78)	0.303	0.0%	0.635

*DNMT1* rs2228611					
GA vs. GG^c^	656/804	1.09 (0.88,1.36)	0.408	0.0%	0.732
AA vs. GG^d^	427/537	0.87 (0.60,1.27)	0.478	11.0%	0.325
GA/AA vs. GG^e^	752/912	1.05 (0.86,1.29)	0.622	0.0%	0.987
AA vs. GA/GG^f^	752/912	0.97 (0.71,1.32)	0.829	56.9%	0.098

*DNMT1* rs8101866					
TC vs. TT^c^	643/1159	0.99 (0.81, 1.21)	0.926	48.2%	0.165
CC vs. TT^d^	411/751	0.80 (0.55,1.17)	0.252	0.0%	0.452
TC/CC vs. TT^e^	686/1255	0.96 (0.80, 1.16)	0.662	0.0%	0.324
CC vs. TC/TT^f^	686/1255	0.80 (0.55,1.17)	0.252	13.1%	0.283

*DNMT3A* rs1550117					
GA vs. GG^c^	839/1548	1.12 (0.93,1.33)	0.229	0.0%	0.436
AA vs. GG^d^	605/1102	**2.03 (1.38,3.00)**	**0.000**	**86.9%**	**0.000**
GA/AA vs. GG^e^	1104/1892	**1.20 (1.01,1.42)**	**0.038**	**69.0%**	**0.040**
AA vs. GA/GG^f^	896/1601	**1.96 (1.33,2.89)**	**0.001**	**85.8%**	**0.001**

*DNMT3A* rs13420827					
CG vs. CC^c^	656/1206	0.84 (0.68,1.03)	0.090	44.3%	0.180
GG vs. CC^d^	495/851	1.16 (0.73,1.85)	0.523	0.0%	0.423
CG/GG vs. CC^e^	689/1255	0.87 (0.72,1.06)	0.171	0.0%	0.336
GG vs. CG/CC^f^	689/1255	1.23 (0.78,1.95)	0.371	0.0%	0.320

*DNMT3B* rs2424913					
CT vs. TT^c^	1086/1053	0.66 (0.32,1.36)	0.258	0.0%	0.992
CC vs. TT^d^	1075/1032	3.02 (0.12,74.69)	0.500	–	–
CT/CC vs. TT^e^	1087/1053	0.71 (0.35,1.44)	0.346	0.0%	0.849
CC vs. CT/TT^f^	1087/1053	3.02 (0.12,74.69)	0.500	–	–

*DNMT3B* rs1569686					
GT vs. TT^c^	745/1262	0.88 (0.69,1.13)	0.320	83.7%	0.002
GG vs. TT^d^	644/1072	0.96 (0.46,2.01)	0.923	3.1%	0.310
GT/GG vs. TT^e^	1225/1789	**0.74 (0.61,0.90)**	**0.003**	**80.1%**	**0.000**
GG vs. GT/TT^f^	756/1283	0.97 (0.46,2.02)	0.930	0.0%	0.394

The bolds pointed to models that had statistically significant associations with gastric cancer.

a *P* value of the Z-test for odds ration test.

b *P* value of the Q-test for heterogeneity test.

c Heterozygote model (heterozygous vs. homozygous frequent allele).

d Homozygote model (homozygous rare vs. homozygous frequent allele).

e Dominant model (homozygous rare + heterozygous vs. homozygous frequent allele).

f Recessive model (homozygous rare vs. heterozygous + homozygous frequent allele).

**Table 3:** Systematic review of associations between *DNMTs* SNPs and gastric cancer risk.

Study	Country	Sample size (cases/controls)	Gene	SNPs	OR (95%CI)	
					Heterozygote model	Homozygote model
Yang et al., 2012	China	242/294	*DNMT1*	rs2114724 C > T	1.16 (0.81, 1.68)	0.62 (0.30, 1.27)
Jiang et al., 2012a, Jiang et al., 2012b	China	447/961	*DNMT1*	rs10420321 A > G	0.96 (0.66, 1.41)	1.17 (1.88,1.55)
Jiang et al., 2012a, Jiang et al., 2012b	China	447/961	*DNMT1*	rs8111085 T > C	1.08 (0.88, 1.43)	1.18 (0.82, 1.69)
Jiang et al., 2012a, Jiang et al., 2012b	China	447/961	*DNMT1*	rs2288349 G > A	0.93 (0.71, 1.22)	0.81 (0.50, 1.33)
Khatami et al., 2009	Iran	200/200	*DNMT1*	rs721186 G > A	1.12 (0.06, 16.0)	–
Khatami et al., 2009	Iran	200/200	*DNMT1*	rs13784 G > A	–	–
Khatami et al., 2009	Iran	200/200	*DNMT1*	rs11488 A > T	–	–
Wu et al., 2012	China	340/251	*DNMT3A*	**rs36012910 A** > **G**	**2.44 (1.37, 4.33)**	1.00 (0.98, 1.01)
Yang et al., 2012	China	242/294	*DNMT3A*	rs13428812 A > G	0.93 (0.64, 1.35)	1.11 (0.58, 2.12)
Yang et al., 2012	China	242/294	*DNMT3A*	rs11887120 T > C	0.96 (0.63, 1.47)	1.26 (0.76, 2.07)
Wu et al., 2014	China	405/408	*DNMT3A*	**rs7560488 T** > **C**	**1.73 (1.24, 2.41)**	**2.50 (1.01, 6.23)**
Wang et al., 2015a, Wang et al., 2015b	China	447/961	*DNMT3B*	rs6119954 G > A	1.00 (0.76, 1.31)	1.37 (0.88, 2.13)
Wang et al., 2015a, Wang et al., 2015b	China	447/961	*DNMT3B*	rs4911107 A > G	0.86 (0.26, 2.88)	0.76 (0.23, 2.46)
Wang et al., 2015a, Wang et al., 2015b	China	447/961	*DNMT3B*	rs4911259 G > T	0.86 (0.26, 2.89)	0.76 (0.23, 2.45)
Wang et al., 2015a, Wang et al., 2015b	China	447/961	*DNMT3B*	rs8118663 A > G	1.28 (0.95, 1.72)	1.32 (0.91, 1.91)
Yang et al., 2012	China	242/294	*DNMT3B*	rs2424908 T > C	0.98 (0.66, 1.45)	1.05 (0.64, 1.71)
Liu, 2008	China	313/350	*DNMT3B*	**rs6087990 C** > **T**	–	**1.46 (1.07, 2.01)**

SNPs, single nucleotide polymorphisms; heterozygote model (heterozygous vs. homozygous frequent allele); homozygote model (homozygous rare vs. homozygous frequent allele).

The bolds pointed to SNPs that had statistically significant associations with gastric cancer.

## Appendix A The Search Strategy for PubMed Comprised the Following

1. Colorectal neoplasms[mesh]
2. DNMT1[tiab]
3. DNMT3A [tiab]
4. DNMT3B[tiab]
5. DNMTs[tiab]
6. DNA methyltransferases[tiab]
7. (2 or 3 or 4 or 5 or 6)
8. 1 and 7

## Appendix A Supplementary data

Table of quality assessment Image 1

Table of Meta-sensitivity analysis Image 2
