# Supplementary material for: DNMT1, DNMT3A and DNMT3B Polymorphisms Associated With Gastric Cancer Risk: A Systematic Review and Meta-analysis
Source: eBioMedicine. 2016 Oct 19;13:125–31. doi: 10.1016/j.ebiom.2016.10.028 (PMC5264435; doi:10.1016/j.ebiom.2016.10.028)
Supplement: Supplementary file 1 — Table of quality assessment [file mmc1.docx]

| Table S1**\|** Score of quality assessment | |
| --- | --- |
| Criteria | Score |
| **Representativeness of case** |  |
| Selected from population cancer registry | 2 |
| Selected from hospital | 1 |
| No method of selection described | 0 |
| **Representativeness of control** |  |
| Population-based | 3 |
| Mixed | 2 |
| Hospital-based | 1 |
| Not described | 0 |
| **Ascertainment of cancer case** |  |
| Histopathologic confirmation | 2 |
| by patient medical record | 1 |
| Not described | 0 |
| **Control selection** |  |
| Controls matched with cases by age and sex | 2 |
| Controls matched with cases only by age or by sex | 1 |
| Not matched or not described | 0 |
| **Genotyping examination** |  |
| Genotyping done blindly and quality control | 2 |
| Only genotyping done blindly or quality control | 1 |
| Not described | 0 |
| **HWE** |  |
| HWE in the control group | 1 |
| HWD in the control group or not mentioned | 0 |
| **Total sample size** |  |
| > 1000 | 3 |
| 501 - 1000 | 2 |
| 201 - 500 | 1 |
| ≤200 | 0 |
| HWE, Hardy-Weinberg equilibrium; HWD, Hardy-Weinberg disequilibrium | |

**Table S2 | Meta-sensitivity analysis of rs1550177 GA/AA vs. GG and rs1569686 GT/GG vs. TT**

| Study omitted | OR (95% CI) | *P_OR_^a^* | *I^2^* | *P_het_^b^* |
| --- | --- | --- | --- | --- |
| ***DNMT3A* rs1550177 GA/AA vs. GG** | |  |  |  |
| Cao (2013) | 1.34 (1.05-1.72) | 0.021 | 79.9% | 0.026 |
| Yang (2012) | 1.26 (1.04-1.54) | 0.018 | 80.9% | 0.022 |
| **Fan (2010)** | **1.06 (0.87-1.29)** | **0.573** | **0.0%** | **0.728** |
| ***DNMT3B* rs1569686 GT/GG vs. TT** | |  |  |  |
| **Wang (2015)** | **0.49 (0.37-0.65)** | **0.000** | **0.0%** | **0.881** |
| Zhang (2014) | 0.74 (0.61-0.91) | 0.004 | 85.0% | 0.000 |
| Hu (2010) | 0.83 (0.67-1.03) | 0.085 | 78.9% | 0.003 |
| Liu (2009) | 0.81 (0.65-1.01) | 0.065 | 82.2% | 0.001 |
| Zhang (2008) | 0.78 (0.64-0.96) | 0.021 | 82.9% | 0.001 |
| ^a^*P* value of the Z-test for odds ration test; ^b^*P* value of the Q-test for heterogeneity test | | | | |
