# Supplementary material for: DNMT1, DNMT3A and DNMT3B Polymorphisms Associated With Gastric Cancer Risk: A Systematic Review and Meta-analysis
Source: eBioMedicine. 2016 Oct 19;13:125–31. doi: 10.1016/j.ebiom.2016.10.028 (PMC5264435; doi:10.1016/j.ebiom.2016.10.028)
Supplement: Supplementary file 2 — Table of Meta-sensitivity analysis [file mmc2.docx]

**
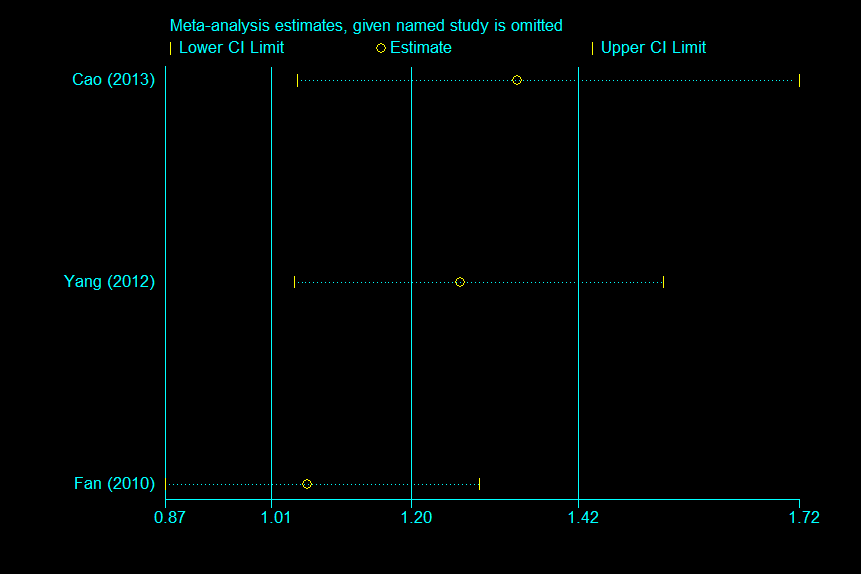
Figure S1: Meta-sensitivity analysis of rs1550177 GA/AA vs. GG**


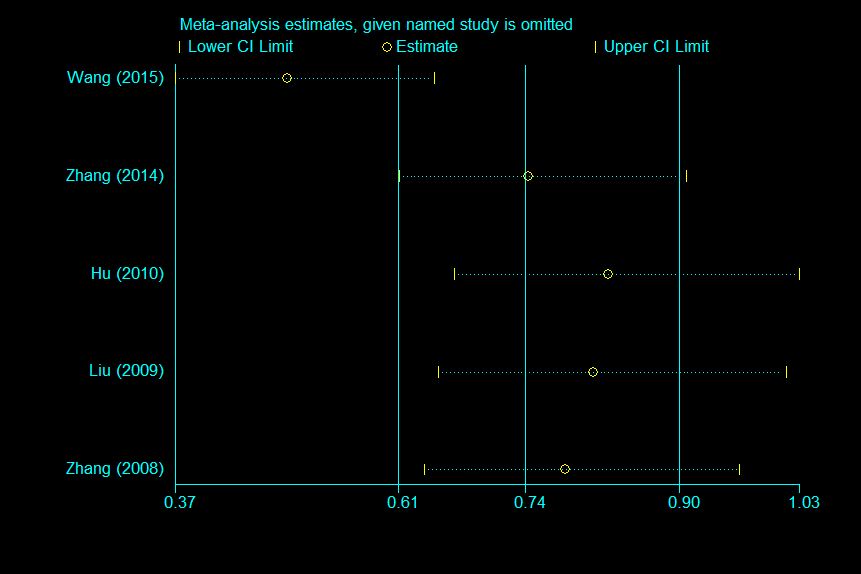
**Figure S2: Meta-sensitivity analysis of rs1569686 GT/GG vs. TT**
